# Regional variations in ex-vivo diffusion tensor anisotropy are associated with cardiomyocyte remodeling in rats after left ventricular pressure overload

**DOI:** 10.1186/s12968-020-00615-1

**Published:** 2020-04-02

**Authors:** Eric D. Carruth, Irvin Teh, Jurgen E. Schneider, Andrew D. McCulloch, Jeffrey H. Omens, Lawrence R. Frank

**Affiliations:** 1 Department of Bioengineering, University of California San Diego, La Jolla, California USA; 2 Leeds Institute of Cardiovascular & Metabolic Medicine, University of Leeds, Leeds, UK; 3 Department of Medicine, University of California San Diego, La Jolla, California USA; 4 Department of Radiology, University of California San Diego, La Jolla, California USA

**Keywords:** Pressure overload hypertrophy, Diffusion tensor imaging, Myocyte remodeling, Transmural gradient

## Abstract

**Background:**

Pressure overload left ventricular (LV) hypertrophy is characterized by increased cardiomyocyte width and ventricle wall thickness, however the regional variation of this remodeling is unclear. Cardiovascular magnetic resonance (CMR) diffusion tensor imaging (DTI) may provide a non-invasive, comprehensive, and geometrically accurate method to detect regional differences in structural remodeling in hypertrophy. We hypothesized that DTI parameters, such as fractional and planar anisotropy, would reflect myocyte remodeling due to pressure overload in a regionally-dependent manner.

**Methods:**

We investigated the regional distributions of myocyte remodeling in rats with or without transverse aortic constriction (TAC) via direct measurement of myocyte dimensions with confocal imaging of thick tissue sections, and correlated myocyte cross-sectional area and other geometric features with parameters of diffusivity from ex-vivo DTI in the same regions of the same hearts.

**Results:**

We observed regional differences in several parameters from DTI between TAC hearts and SHAM controls. Consistent with previous studies, helix angles from DTI correlated strongly with those measured directly from histological sections (*p* < 0.001, R^2^ = 0.71). There was a transmural gradient in myocyte cross-sectional area in SHAM hearts that was diminished in the TAC group. We also found several regions of significantly altered DTI parameters in TAC LV compared to SHAM, especially in myocyte sheet angle dispersion and planar anisotropy. Among others, these parameters correlated significantly with directly measured myocyte aspect ratios.

**Conclusions:**

These results show that structural remodeling in pressure overload LV hypertrophy is regionally heterogeneous, especially transmurally, with a greater degree of remodeling in the sub-endocardium compared to the sub-epicardium. Additionally, several parameters derived from DTI correlated significantly with measurements of myocyte geometry from direct measurement in histological sections. We suggest that DTI may provide a non-invasive, comprehensive method to detect regional structural myocyte LV remodeling during disease.

## Background

Systemic hypertension is a major health challenge, affecting approximately 45% of adults in the United States based on current guidelines [1]. The well-known response of the left ventricle (LV) to such pressure overload is concentric hypertrophy, which is characterized by the addition of sarcomeres in parallel within cardiomyocytes, resulting in LV wall thickening without chamber dilation. Such remodeling in pressure overload hypertrophy is considered a compensatory mechanism to maintain cardiac output despite increased downstream resistance to blood flow. This compensatory state, however, often leads to impaired diastolic filling, accompanied by wall thinning, chamber dilation, impaired systolic function, and eventual heart failure [2, 3].

Many studies have described effects of pressure overload on structural features, e.g. cardiomyocyte hypertrophy, laminar cardiomyocyte sheet architecture, proteins (both myocyte and extracellular matrix), molecular signaling, and gene expression networks [4–9]. However, it is unclear whether remodeling of structural features in the heart during pressure overload hypertrophy, such as fibrosis and myocyte hypertrophy, occurs uniformly in all LV regions. Spatial gradients of structure and mechanical function are found in the normal and diseased LV, especially transmurally [10]. These transmural gradients, together with other factors, are thought to contribute to normalizing the otherwise large gradients in stress and strain along the long-axis of the myocytes (sometimes referred to as “fiber” stress or strain) during both diastolic filling and systolic contraction [10–15].

For example, a paper on myocyte dimensions reported a transmural gradient in myocyte geometry in isolated cells from normal hearts, with larger myocyte volume and cross-sectional area (CSA) in the sub-endocardium (ENDO) compared to the midwall (MID) or sub-epicardium (EPI) [16]. In pressure overload hypertrophy, this transmural gradient was “abolished” [17]. However, others have shown different transmural patterns of myocyte geometry in both normal and hypertrophied rat LV [18, 19]. These conflicting results could be due to differences in loading conditions, different strains of rat, experimental processes such as fixation, or other factors. The primary goal of this study was to improve characterization of the transmural remodeling response to pressure overload by combined assessment of myocyte cell hypertrophy and sheet structure.

Measuring variations in these structural features is not trivial and usually involves histological methods, including tissue sectioning, staining, imaging, and careful measurement. Not only is this time-consuming and tedious, but such experiments cannot be performed in vivo, are destructive, and are usually only performed in small sub-regions of an organ or tissue. Non-destructive imaging is a much more appealing approach, especially methods which can relatively quickly and accurately capture the information in the entire organ or tissue of interest, while preserving 3D geometry. Cardiovascular magnetic resonance (CMR) diffusion tensor imaging (DTI) has emerged as one such technique to measure local structural orientations from eigenvectors, such as the local myocyte orientation described by the “helix” or “fiber” angle, and local laminar sheet orientations [20–23]. The diffusion tensor not only provides orientation information in its eigenvectors, but also the apparent diffusivities along those eigenvectors in the eigenvalues. Some recent studies have described transmural variations in fractional anisotropy (FA), which is derived from the eigenvalues and indicates the degree of self-diffusion anisotropy [24–26]. Others have shown an increase in secondary and tertiary diffusivities and sheet reorientation in heart failure patients [27, 28]. These results are also somewhat conflicting and unclear, and even less well understood are the contributions of different structural features to variations in 3D diffusivity. Thus, a secondary objective of this study was to determine whether regional and pathophysiological variations in these structural features could be detected consistently using DTI.

## Methods

### Pressure overload hypertrophy model and tissue preparation

All animal studies were approved by the Institutional Animal Care and Use Committee at UCSD and followed the NIH Guide for the Care and Use of Laboratory Animals. Sixteen male Sprague-Dawley rats weighing 206 ± 12 g were randomly divided equally into transverse aortic constriction (TAC) and SHAM groups. Rats were sedated in a chamber with 5% isoflurane in air, intubated, and anesthetized for the duration of the surgery with 1.25–2.00% isoflurane in air. An appropriate level of anesthesia was confirmed by toe pinch. Once sufficiently anesthetized, a small incision was made to expose the ascending aorta. In TAC animals, a hemo-clip was inserted between the brachiocephalic and left carotid arteries, then constricted to a fixed width of 0.5 mm, which constricts the aortic lumen to approximately 50–60% of its original cross-sectional area. SHAM controls underwent the same procedure but did not have the clip inserted. The incision layers were closed and sutured, and the animal was allowed to fully recover.

M-mode echocardiography was performed in isoflurane-sedated rats 1 day before surgery, and at 2 weeks and 4 weeks after surgery to obtain ventricle dimensions and volumes (Table 1). At 4 weeks, manometer (Millar, Houston, Texas, USA) catheter recordings of aortic (proximal to the clip) and LV cavity pressures as well as concurrent electrocardiograms were also recorded [29]. Hearts were then excised, weighed, and retrograde perfused with Krebs-Henseleit buffer until the tissue was cleared of blood and regular beating resumed. Cardiac arrest in the relaxed state was achieved using a modified Krebs-Henseleit buffer with high potassium, and hearts were then fixed in this relaxed state using Karnovsky’s fixative with 2 mM gadolinium chelate (4.4 mL of 0.5 M ProHance (ProHance; Bracco, Eden Prairie, Minnesota, USA) was added to make a 1 L solution, 0.44% by volume). For imaging, hearts were embedded in a 1% agarose gel containing 2 mM gadolinium chelate. A subset of these hearts was used in a previously published imaging study [30].

**Table 1:** Compensated hypertrophy established in TAC group at 4 weeks

Variable [units]	Week 0		Week 4		
	SHAM	TAC	SHAM	TAC	*p*-value
ED IVS [mm]	0.98 ± 0.02	0.98 ± 0.03	1.15 ± 0.02	1.47 ± 0.03	< 0.001*
ED LVID [mm]	7.03 ± 0.12	6.90 ± 0.13	7.80 ± 0.19	8.13 ± 0.22	0.30
ED LVPW [mm]	1.33 ± 0.02	1.38 ± 0.04	1.50 ± 0.07	2.00 ± 0.12	< 0.01*
ED AoP [mmHg]	–	–	85 ± 2	77 ± 4	0.11
ES IVS [mm]	1.36 ± 0.06	1.29 ± 0.06	1.51 ± 0.10	1.80 ± 0.11	0.10
ES LVID [mm]	3.93 ± 0.07	3.86 ± 0.09	4.45 ± 0.15	5.09 ± 0.21	< 0.05*
ES LVPW [mm]	2.32 ± 0.06	2.21 ± 0.05	2.53 ± 0.10	2.94 ± 0.12	< 0.05*
ES AoP [mmHg]	–	–	118 ± 3	186 ± 11	< 0.001*
FS [%]	44 ± 1	44 ± 1	43 ± 1	37 ± 1	< 0.01*
EF [%]	81 ± 1	81 ± 1	79 ± 1	72 ± 2	< 0.01*
CO [L/min]	0.22 ± 0.01	0.22 ± 0.01	0.29 ± 0.02	0.28 ± 0.02	0.92
BW [g]	209 ± 5	204 ± 4	334 ± 8	324 ± 6	0.36
HW [g]	–	–	1.14 ± 0.02	1.59 ± 0.04	< 0.001*
HW/BW [g/kg]	–	–	3.42 ± 0.05	4.91 ± 0.15	< 0.001*

Structural and functional measurements before and at 4 weeks post-surgery in TAC and SHAM groups. All measurements are from echocardiograms, with the exception of AoP, BW, HW, and HW/BW, which were measured at the time of excision. *Abbreviations*: *ED* end-diastole, *ES* end-systole, *IVS* interventricular septum thickness, *LVID* left ventricular internal diameter, *LVPW* left ventricular posterior wall thickness, *AoP* aortic pressure, *FS* fractional shortening, *EF* ejection fraction, *CO* cardiac output, *BW* body weight, *HW* wet heart weight. *p < 0.05

### Diffusion tensor CMR acquisition

Images were acquired using a fast spin echo sequence similar to previously published data, with some modification [31]. Briefly, a 9.4 T CMR magnet (Agilent Technologies, Santa Clara, California, USA) capable of a maximum gradient strength of 1 T/m was used, together with a 20 mm inner diameter birdcage coil (Rapid Biomedical, Rimpar, Germany). The TR was specified at 250 ms to maximize signal-to-noise (SNR) efficiency, and TE was set to a minimum (9.3 ms). Other imaging parameters were as follows: echo spacing = 4.9 ms, echo train length = 8, FOV = 21.6 × 14.4 × 14.4 mm^3^, resolution = 100 × 100 × 100 μm^3^, number of diffusion weighted (DW) directions = 30 forward + 30 reverse, number of non-DW (b = 0) images = 4, b_effective_ = 1000 s/mm^2^ [32], diffusion gradient duration (δ) = 2 ms, diffusion time (Δ) = 5.5 ms. Total acquisition time per heart was 11.5 h.

### Tissue sectioning, staining, and confocal microscopy

After DTI, whole heart samples (*n* = 4 per group) were dissected into three sections after removal of the atria and valve plane: the right ventricle, interventricular septum, and LV free wall. Samples from the equatorial LV free wall were dehydrated in graded ethanol and embedded in paraffin wax for sectioning. Serial sections at 50 μm thickness were obtained parallel to the epicardial surface through the entire ventricular wall using a microtome and mounted on slides. The staining protocol was similar to that described previously, but without the DAPI (nucleus) stain [33]. After wax removal and rehydration, tissue sections were stained with wheat germ agglutinin (WGA) conjugated to Alexa Fluor 633 (Life Technologies, Carlsbad, California, USA) to label the interstitial space. A Zeiss LSM 880 with Airyscan FAST (Zeiss, Jena, Germany) was used to acquire image volumes in FAST mode. Image stacks were acquired with the following settings: Objective = 20x Pln/Apo, numerical aperature = 0.8, resolution = 0.2 × 0.2 × 0.4 μm^3^, typical image size = 1220 × 1220 × (80–130) voxels.

Bright-field images were also obtained of the same sections at low magnification (10X) on an EVOS FL Auto Microscope (Life Technologies, Thermo Fisher Scientific, Waltham, Massachusetts, USA) to measure helix angles and dispersions. Helix angles and dispersions were measured using the image gradient technique described previously [34] and implemented in-house in MATLAB (The MathWorks, Natick, Massachusetts, USA). The helix angle and dispersion for a given section were defined as the circular mean and circular standard deviation of detected edges.

### Image processing, registration, mesh generation, angle/parameter calculation

The SNR for each heart was defined as

$$ SNR=\frac{{\overline{I}}_{sig}}{\sigma_{bkg}} $$

where ${\overline{I}}_{sig}$ is the mean intensity of voxels in a region of myocardium, and *σ*_*bkg*_ is the standard deviation of voxel intensities in a region of background signal. Diffusion tensors were calculated from diffusion weighted images in AFNI [35] by solving the standard monoexponential, Gaussian model:

$$ \frac{S\left(\boldsymbol{b}\right)}{S(0)}=\exp \left(-\boldsymbol{bD}\right) $$

where *b* is the b-value [s/mm^2^] indicating the combined magnitude, separation, and duration of the diffusion gradient, *S*(*b*) is the diffusion-weighted image signal, *S*(0) is the non-weighted image (b = 0), and *D* is the apparent diffusion coefficient [mm^2^/s].

**Fig. 1:**
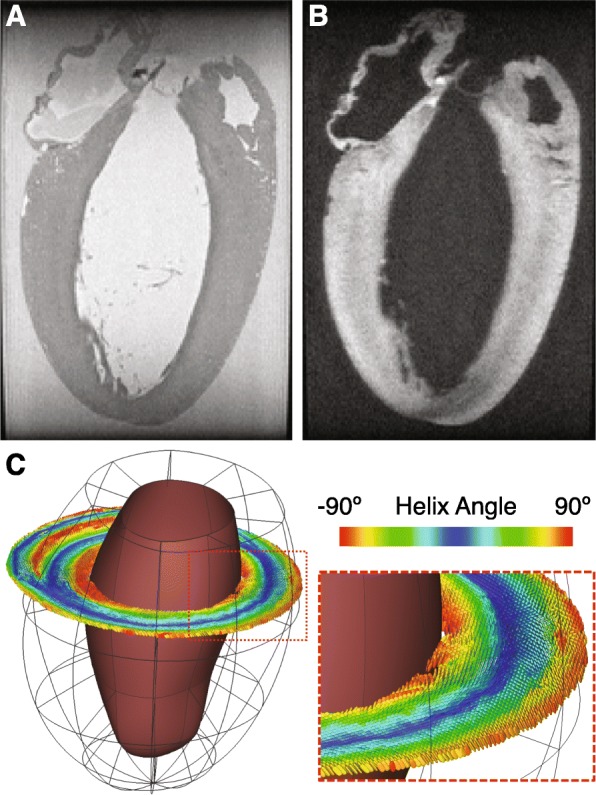
Diffusion weighted images and registration. **a** Representative b = 0 image (i.e. no diffusion weighting). Signal-to-noise (SNR) was roughly 33 ± 1 across all animals. **b** Diffusion weighted image of the same slice as in (**a**). SNR was roughly 24 ± 1 across all animals. **c** Rendered diffusion tensors from a single short axis slice superimposed on the corresponding finite element mesh

Masks were generated by semi-automatic segmentation of the LV from b = 0 images in Seg3D (SCI Institute, University of Utah, Salt Lake City, Utah, USA). Papillary muscles and trabeculae were excluded from the segmentation. Segmented masks were converted into mesh surfaces, which were then separated into endocardial and epicardial surfaces. An affine transformation was applied to align each data set with a consistent “cardiac” reference frame using the long axis of the LV and the axis bisecting the right ventricle. The same affine transformation was applied to the calculated diffusion tensors using the log-Euclidian transform to preserve tensor properties [36]. A prolate spheroidal mesh template with 4 longitudinal and 10 circumferential (40 total) elements was fitted via least-squares to the LV endocardial and epicardial surfaces. This mesh served as a consistent datum across all hearts by which to measure local helix and sheet angles from the eigenvectors, and to simply and consistently subdivide the tensor data by anatomical region (circumferential, longitudinal, and transmural position) for direct regional comparisons across all hearts. An example mesh with rendered diffusion tensors in a single image slice are shown in Fig. 1c.

The primary, secondary, and tertiary eigenvectors from DTI have been shown to agree closely with the average local myocyte long-axis, sheet, and sheet-normal orientations, respectively, and the eigenvalues represent the apparent diffusion coefficients along those principal directions [20–22]. Helix and sheet angles were calculated as previously described [37]. Briefly, the helix angle was defined as the angle between the local circumferential direction and the projection of the primary eigenvector onto the circumferential-longitudinal plane. The sheet angle was defined as the angle between the local radial direction and the secondary eigenvector. The dispersions of these angles were defined as the circular standard deviation of the angles in each spatial region. The mean diffusivity (MD) is the mean of the three eigenvalues. Fractional (FA), linear (CL), planar (CP), and spherical (CS) anisotropies were calculated from the eigenvalues, and their mean ($\hat{\lambda}$) as described previously [38]:

$$ FA=\sqrt{\frac{3}{2}}\sqrt{\frac{{\left({\lambda}_1-\hat{\lambda}\right)}^2+{\left({\lambda}_2-\hat{\lambda}\right)}^2+{\left({\lambda}_3-\hat{\lambda}\right)}^2}{\left({\lambda}_1^2+{\lambda}_2^2+{\lambda}_3^2\right)}\ } $$

$$ CL=\frac{\lambda_1-{\lambda}_2}{3\hat{\lambda}} $$

$$ CP=\frac{2\left({\lambda}_2-{\lambda}_3\right)}{3\hat{\lambda}} $$

$$ CS=\frac{\lambda_3}{\hat{\lambda}} $$

For a more detailed explanation of these values, we refer the reader elsewhere (see Fig. 1) [39].

Confocal images were processed using Zeiss Airyscan Processing. Measurements were made of myocyte length, in-plane (parallel to the epicardial surface) myocyte width, and through-plane myocyte height from over 350 myocytes at various wall depths. Lengths were defined as the longest dimension of the myocyte. Width and height were measured across visible nuclei, or across the largest cross-section if no nucleus was visible. Aspect ratios were also computed for length:width ratio (L:W), length:height (L:H), and width-height (W:H). CSA was computed assuming an elliptical cross-section, with major and minor radii defined as half the width and height, respectively. Additionally, myocyte fractional anisotropy (MFA), analogous to FA from DTI, was calculated as:

$$ MFA=\sqrt{\frac{3}{2}}\sqrt{\frac{{\left(l-\hat{d}\right)}^2+{\left(w-\hat{d}\right)}^2+{\left(h-\hat{d}\right)}^2}{\left({l}^2+{w}^2+{h}^2\right)}\ } $$

where $\hat{d}$ is the mean of myocyte length (*l*), width (*w*), and height (*h*).

All descriptive statistics in this work comparing SHAM and TAC groups are reported as mean ± standard error of the mean. For all statistical inference tests, *p*-values < 0.05 were considered statistically significant. Student’s unpaired, two-tailed t-test with assumed unequal variances was used to test for significant differences between TAC and SHAM parameters, such as heart weight to body weight ratio (HW/BW) and ejection fraction (EF).

The DTI data from each LV were divided into 600 anatomically registered regions (20 transmurally, 10 circumferentially, and 3 longitudinally). A multi-factor ANOVA for all variables was conducted, with surgical intervention, transmural, circumferential, and longitudinal positions as the four factors. Only between-group differences and the direct linear interactions between factors were considered. Correction for multiple comparisons was performed using the Tukey-Kramer method. For visualization, DTI data were divided into 5400 regions (20 transmurally, 30 circumferentially, 9 longitudinally). Group means and the normalized difference between groups (analogous to a t-score) was calculated for each of the 5400 regions. Maps of these values are shown in Figs. 3, 4.

For correlations between parameters measured from DTI and measurements from microscope images, Pearson’s correlation coefficient was calculated, along with the *p*-value for the linear correlation. A random permutation test with 10,000 permutations was performed to correct for multiple comparisons. Once significant correlations were found, analysis of covariance (ANCOVA) was applied to determine the relative contributions to correlation from the SHAM and TAC groups.

## Results

### Transverse aortic constriction and left ventricular hypertrophy

At four weeks post-surgery, the systolic pressures in the ascending aorta were significantly elevated in the TAC group compared to SHAM controls (186 ± 33 mmHg TAC vs 118 ± 9 mmHg SHAM, *p* < 0.01). Animals in the TAC group were found to be in a state of compensated hypertrophy, with significantly higher HW to BW ratio (4.91 ± 0.45 g/kg TAC vs 3.42 ± 0.16 g/kg SHAM, *p* < 0.001), and a significantly lower, yet still normal (i.e. > 50%) EF (79 ± 1% SHAM vs. 72 ± 2% TAC, p < 0.01). Table 1 summarizes the key differences found between SHAM and TAC hearts.

### Diffusion tensor CMR

**Fig. 2:**
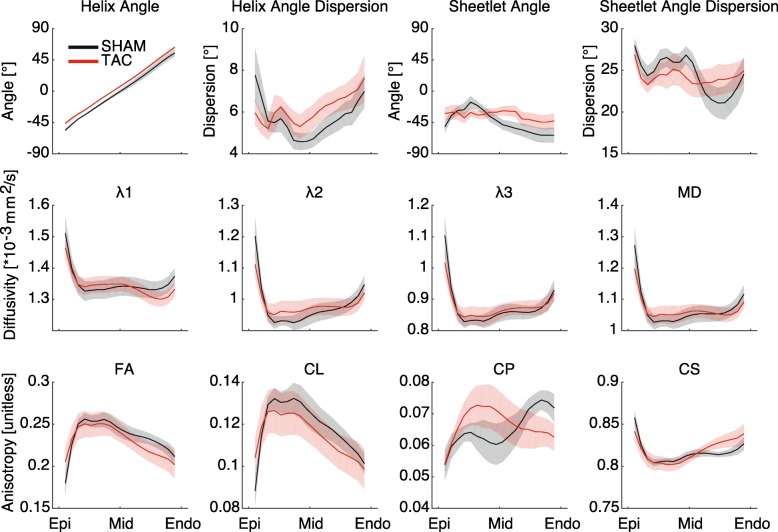
Transmural gradients of diffusion tensor (DT) parameters in equatorial LV free wall. Of note is the slight increase in helix angle at all points across the wall in transverse aortic constriction (TAC) vs SHAM. Transmural differences in planar anisotropy and sheet angle dispersion are apparent in this region between TAC and SHAM

**Fig. 3:**
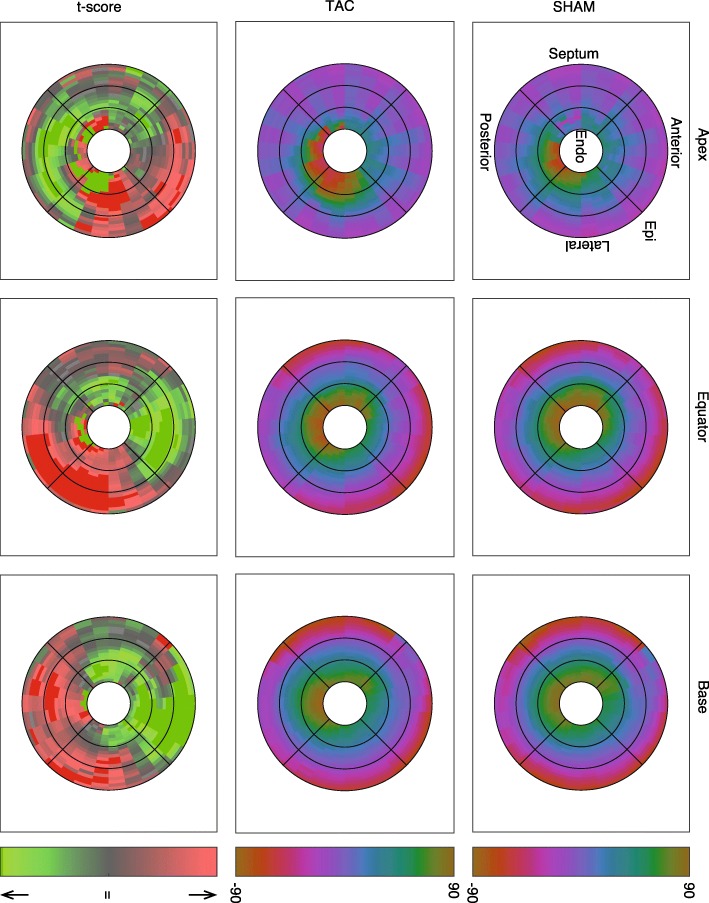
Regional variation in helix angle. The right column is the mean of all SHAM rats, the middle column is mean of the TAC group, and the left column is the calculated t-score between TAC and SHAM groups. The rows represent the longitudinal location of each slice, and the radial position of each plot represents transmural location. In the t-score plot, red indicates TAC > SHAM, green indicates TAC < SHAM, with bright colors indicating a t-score that would give rise to an uncorrected p-value < 0.05. Note the increase in helix angle in the equatorial region of the LV free wall, and lowered helix angles in the anterior wall

The mean (± standard deviation) SNR of the b = 0 images across all hearts was 33 ± 4. In diffusion weighted images, the mean SNR across all animals was 24 ± 4. Representative b = 0 and DW images are displayed in Fig. 1a, b. Figure 2 shows the transmural distributions of the 12 major variables discussed in this work in the equatorial LV lateral wall. Notably, the helix angles in this region were consistently slightly higher in TAC animals compared to SHAM controls by approximately 7–8 degrees at all points across the wall, whereas in the anterior region of the equator and base, the opposite was true. In the septal wall, helix angles were more similar between TAC and SHAM groups across the wall (Fig. 3). Also of interest is the transmural variation in CP and sheet angle dispersion.

**Fig. 4:**
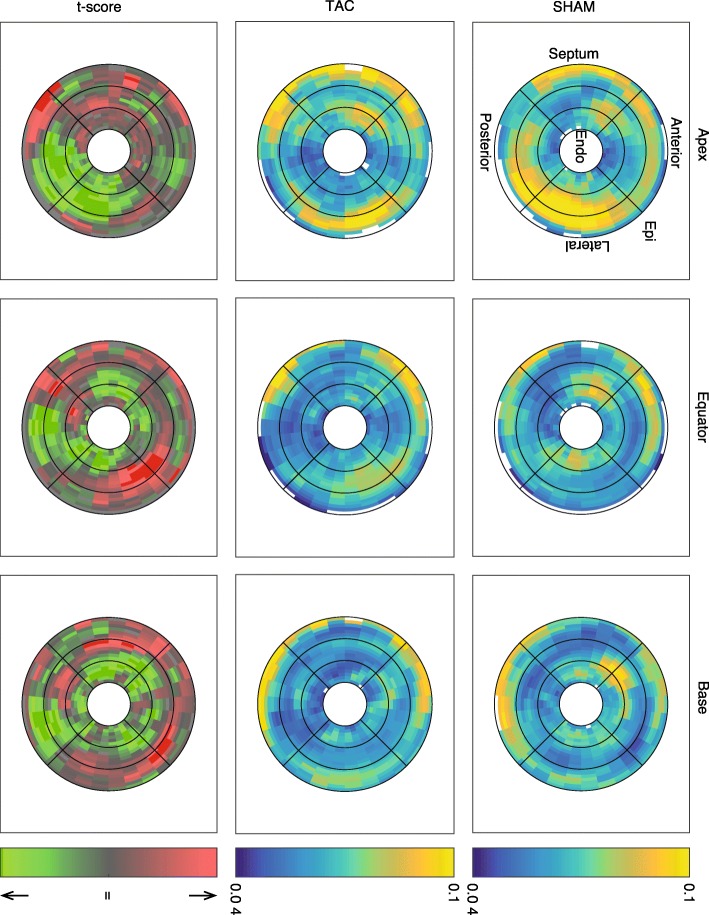
Regional variation in planar anisotropy (CP). The right column is the mean of all SHAM rats, the middle column is mean of TAC group, and the left column is the calculated t-score between TAC and SHAM groups. The rows represent the longitudinal location of each slice, and the radial position of each plot represents transmural location. In the t-score plot, red indicates TAC > SHAM, with bright red indicating a t-score that would give rise to an uncorrected *p*-value < 0.05. Planar anisotropy was generally lower in sub-endocardial regions in TAC hearts compared to SHAM, but slightly higher in the sub-epicardium

The regional analysis of diffusion tensor-derived parameters produced several other interesting results, which are summarized in Table 2. Regional variations in some key variables of SHAM and TAC hearts, as well as the normalized difference between the groups, are shown in Figs. 3, 4. The main differences found between SHAM and TAC groups were as follows: The helix angle was significantly different in both the mean and the interactions transmurally, circumferentially, and longitudinally, and was apparently slightly higher in the posterolateral EPI (i.e. more circumferential), and slightly lower in the anterior ENDO (also more circumferential) in TAC vs SHAM. The sheet angle dispersion was lower on average in TAC hearts compared to SHAM, and the interactions with transmural, circumferential, and longitudinal position were also significant. Although the diffusivities alone did not show much collective significance in the ANOVA, there was an apparent transmural-longitudinal shift in all three, with lower diffusivities in ENDO and apical regions in TAC vs SHAM. There was a notable transmural gradient of FA in both SHAM and TAC hearts, and this value appeared to decrease in the ENDO and apical regions, whereas it was increased elsewhere in TAC hearts.

**Table 2:** ANOVAs for DTI parameter regional variation (600 regions) corrected for multiple comparisons

Variable	Hypertrophy	Hyp*Transm	Hyp*Circm	Hyp*Long
Helix Angle	< 0.001*	< 0.001*	< 0.001*	< 0.01*
Helix Angle Dispersion	< 0.001*	< 0.001*	< 0.01*	< 0.001*
Sheet Angle	< 0.01*	0.19	< 0.001*	< 0.001*
Sheet Angle Dispersion	< 0.001*	< 0.01*	< 0.05*	< 0.001*
Primary Eigenvalue (*λ*_1_)	0.559	–	–	–
Secondary Eigenvalue (*λ*_2_)	0.449	–	–	–
Tertiary Eigenvalue (*λ*_3_)	< 0.05*	< 0.05*	0.568	0.994
MD ($\hat{\lambda}$)	0.187	–	–	–
FA	< 0.01*	< 0.001*	0.697	< 0.001*
CL	0.388	–	–	–
CP	< 0.001*	< 0.001*	0.89	0.24
CS	< 0.001*	< 0.001*	0.75	< 0.001*

*Abbreviations*: *MD* mean diffusivity, *FA* fractional anisotropy, *CL* linear anisotropy, *CP* planar anisotropy, *CS* spherical anisotropy. *p < 0.05

### Confocal micrographs

**Fig. 5:**
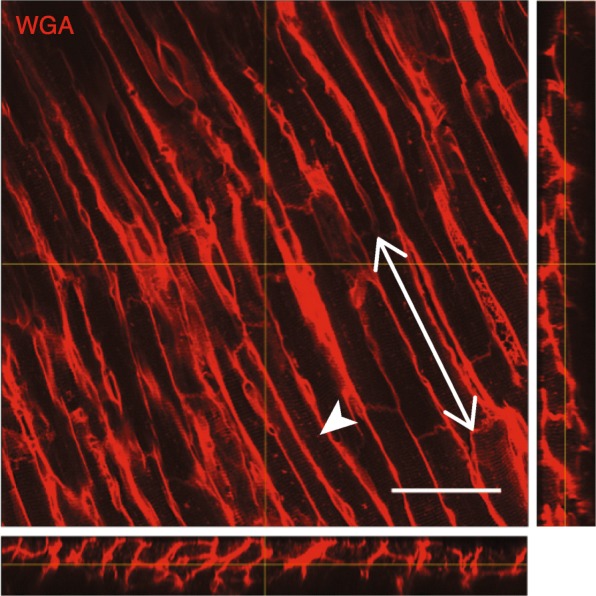
Orthogonal views of a representative confocal image volume. The extracellular space is clearly visible with the wheat germ agglutinin (WGA) stain (red), including some t-tubule patterns (arrowhead). Cell ends are also visible, making measurement of myocyte length possible, as shown for example by the arrow. Out-of-plane dimension can also readily be measured in orthogonal views. Scale bar: 50 μm

**Fig. 6:**
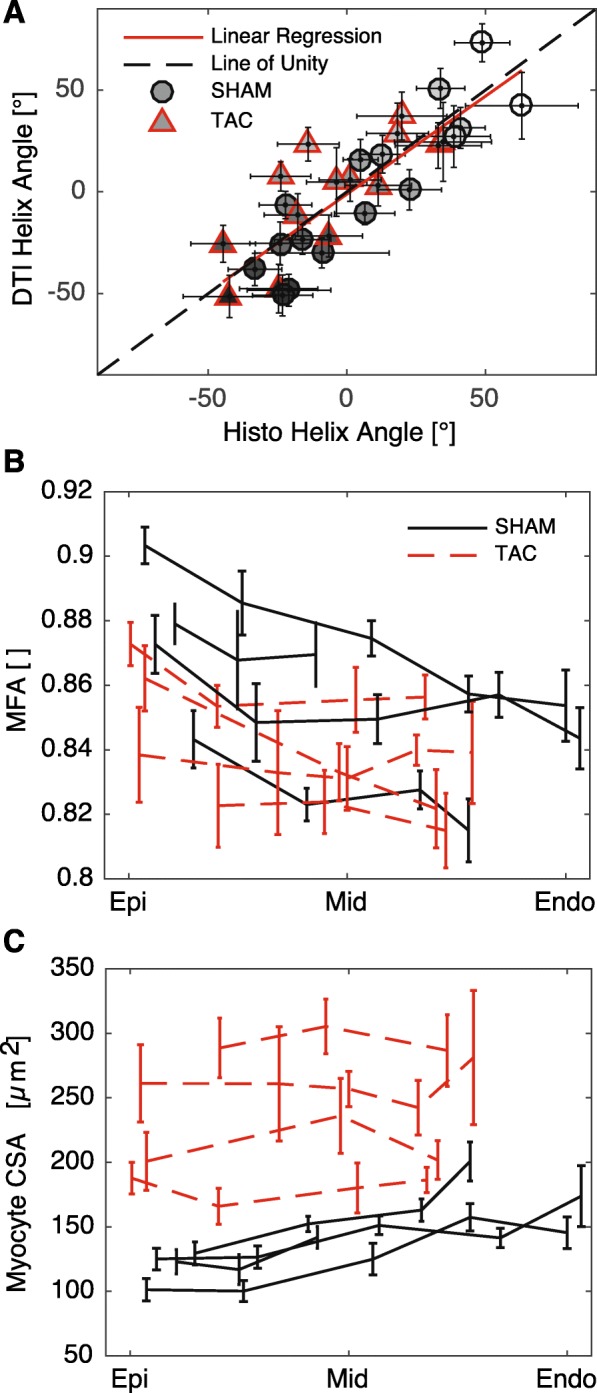
Summary of myocyte measurements from histological sections. Data are presented as mean ± SD of measurements in each region for SHAM (*n* = 4) and TAC (n = 4) groups. **a** As expected, helix angles from histological sections correlated strongly (R^2^ = 0.71, *p* < 0.001) with those from DTI. Grayscale color of markers indicates transmural location (black: ENDO, white: EPI). **b** A transmural gradient in myocyte fractional anisotropy (MFA) was observed, with lower values in the ENDO than EPI, especially in SHAM myocytes. **c** Myocyte cross sectional area (CSA) also exhibited a transmural gradient in SHAM hearts, with larger values in the ENDO. In TAC hearts, CSA was elevated, and the transmural gradient was diminished

A representative image volume from confocal imaging is shown in three orthogonal views in Fig. 5. Lateral and longitudinal cell borders are clearly distinguishable, and t-tubules are also visible. Transmural plots of CSA and MFA are shown in Fig. 6. As expected, myocytes in TAC hearts exhibited a larger CSA compared to SHAM controls. Additionally, we observed a transmural gradient trend in myocyte CSA in SHAM hearts, which tended to increase slightly from EPI to ENDO. The transmural gradient in CSA appeared diminished in TAC hearts, similar to previously measured data [17]. However, better histological coverage across the wall and larger sample sizes are needed to verify these transmural patterns.

### Correlations between DTI and Myocyte geometry

**Fig. 7:**
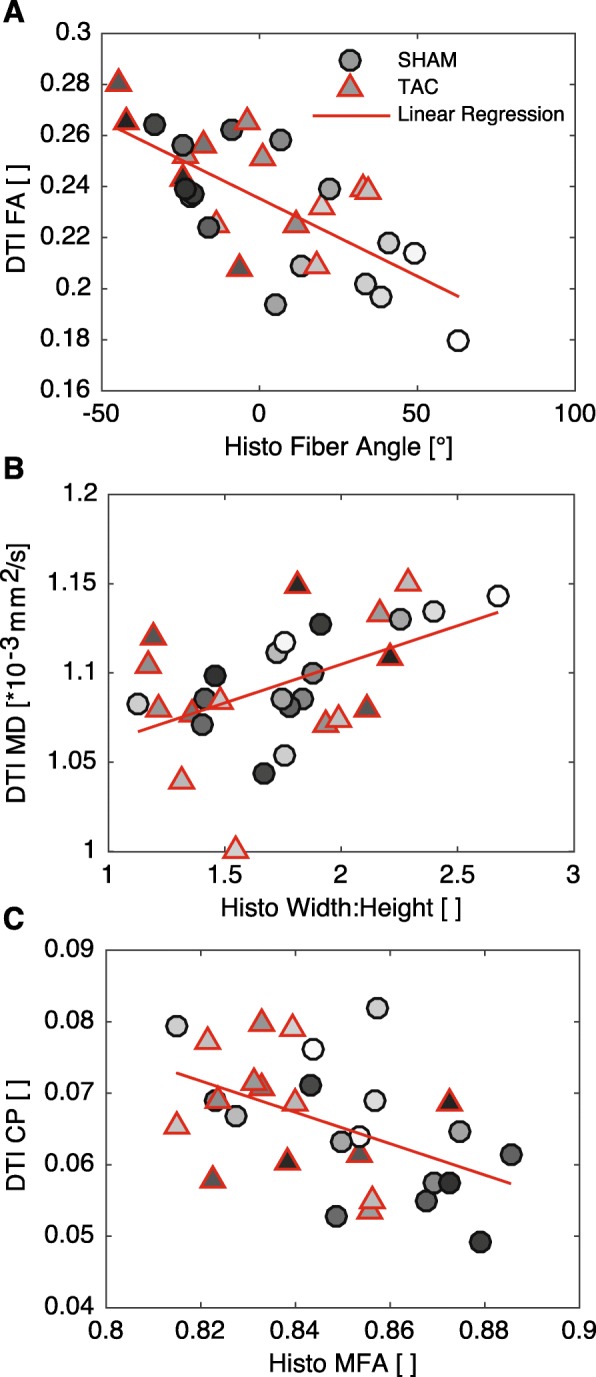
Correlations between select DTI-derived parameters (ordinate) and measurements from histological sections (abscissa). Grayscale color of markers indicates transmural location (black: ENDO, white: EPI). **a** Fractional anisotropy from DTI exhibited a strong correlation with helix angle (R^2^ = 0.48, p < 0.001). **b** Mean diffusivity correlated with myocyte width:height ratio (R^2^ = 0.25, *p* < 0.01), and **c** Planar anisotropy correlated negatively with MFA (R^2^ = 0.24, p < 0.01)

Several significant correlations were found between the DTI data myocyte geometry. As expected, helix angles measured from bright field images and from DTI correlated significantly (*p* < 0.001, R^2^ = 0.71; Fig. 6a). Helix angles from DTI also correlated with L:W ratio (*p* < 0.01, R^2^ = 0.23), indicating that myocyte geometry varies transmurally. Additional significant correlations between DTI-derived parameters and those measured from confocal images of tissue sections were as follows: The primary eigenvalue diffusivity (*λ*_1_) and all anisotropy metrics correlated significantly with helix angle (p < 0.01, R^2^ = 0.27), again, indicating a transmural gradient. Planar anisotropy correlated significantly with helix angle (p < 0.01, R^2^ = 0.27), myocyte length:width ratio (p < 0.01, R^2^ = 0.24), and MFA (p < 0.01, R^2^ = 0.24. The results of ANCOVA tests are presented in Table 3. Three of these correlations are also plotted in Fig. 7.

**Table 3:** ANCOVAs for significant correlations

DTI Parameter	Histo Parameter	^a^Best ANCOVA	R^2^	Correlation *p*-value	ANCOVA ***p***-value(s)
Helix Angle	Helix Angle	Same Line	0.71	<< 0.001 (6e-9)	
	L:W	Same Line	0.23	0.0073	
Helix Angle Dispersion	Helix Angle	Parallel Lines	0.23	0.0078	0.0063/0.0008
	Myocyte Width	Same Line	0.31	0.0013	
	L:W	Separate Lines	0.30	0.0019	0.0363/0.0046
Sheet Angle Dispersion	Helix Angle	Same Line	0.20	0.0130	
	L:H	Same Line	0.25	0.0049	
Primary Eigenvalue	Helix Angle	Separate Lines	0.27	0.0036	0.0121/0.0025
Secondary Eigenvalue	W:H	Same Line	0.21	0.0112	
MD	W:H	Same Line	0.25	0.0053	
FA	Helix Angle	Same Line	0.48	< 0.001 (2e-5)	
CL	Helix Angle	Same Line	0.56	< 0.001 (2e-6)	
CP	Helix Angle	Same Line	0.27	0.0032	
	L:W	Same Line	0.24	0.0062	
	MFA	Same Line	0.24	0.0057	
CS	Helix Angle	Same Line	0.26	0.0038	

^a^Best ANCOVA indicates whether there is a different spatial relationship between SHAM and TAC groups. Same lines indicate that the DTI parameter and histological measurement are associated, regardless of TAC or SHAM group (i.e. correlation); parallel lines indicate a similar spatial relationship between variables for both groups, but with an offset; separate lines indicate a different spatial relationship between groups

## Discussion

In this work, we have shown that several microstructural features in ex-vivo rat myocardium, including myocyte aspect ratios, correlated significantly with parameters derived from DTI in the same regions of the same hearts. Most of these correlations held true regardless of surgical group (TAC vs SHAM), whereas the association of others were modulated by pressure overload (Table 3). Our results also may support the conclusion that transmural gradients in myocyte geometry, including CSA and aspect ratios, are present in the normal rat LV but are reduced in pressure overload hypertrophy. This normal transmural gradient has been characterized by larger myocyte CSA in ENDO than EPI [17]. A similar trend appears to be present in our data, however, further study with larger sample sizes is required to confirm this pattern and its association with DTI.

We also observed a difference in the transmural patterns of several other structural features, as measured from DTI. One notable example is the orientation and dispersion of sheet angles. In agreement with our hypothesis, we observed that the transmural gradients in both the mean sheet angle and the dispersion of those angles about the mean, were more transmurally uniform in the LV of rats with pressure overload hypertrophy. This does not indicate that sheet angles are globally reoriented to become, for example, more radially aligned as others have observed [28]. Rather, transmural variation is reduced so that across the wall, the orientation and dispersion of sheet structures were more similar. Also of note, CP varied in the equatorial LV free wall, with a different pattern in SHAM than TAC hearts. In this region, CP decreased from EPI to ENDO in SHAM hearts, with a low point in MID, whereas in TAC hearts, CP was lower in EPI and ENDO regions, with a high point in MID (Fig. 2). Given that CP correlated negatively with MFA and L:W ratio, it may be that CP is altered (increased) in hypertrophied myocytes, due to anisotropic fibrosis or cleavage plane remodeling.

Others have reported transmural gradients and responses to pressure overload hypertrophyin the rat LV with different patterns than the present study [18, 19]. There are many factors that could contribute to these differences, including rat strain, the method of inducing pressure overload, and differences in measurement methods. However, our results appear to agree with those of Campbell et al., who showed a transmural gradient in myocyte CSA which is normalized in pressure overload hypertrophyin the rat [16, 17]. Others have reported that transmural gradients in mitochondrial respiratory chain activity and oxidative stress are normalized in pressure overload hypertrophy [40]. As sarcomeres are added and myocyte volume increases, the metabolic capacity must also increase to facilitate the energetic demands of those additional sarcomeres. It is clear that a remodeling balance must be achieved between the need to respond to the increase in downstream resistance to blood flow and the mechanical optimization of maintaining uniform myocyte long-axis stress and strain under such conditions. Several structural features have been shown to exhibit a transmural gradient in normal tissue, but remodel during pressure overload hypertrophy, which may be associated with the observed changes presented here [10].

Although in its infancy, the in vivo application of DTI in human hearts has already begun to show promise for detecting structural features in healthy tissue and in remodeled tissue during disease [28, 41–43]. If our approach were to be applied in vivo and/or to other species, several points would be important to consider. For example, our results, especially regarding helix and sheet angle dispersions, may depend strongly on image resolution, which required long scan times (> 11 h) at high field strengths to achieve the high-fidelity DTI data presented here. Additionally, differences between species, e.g. perfusion, may impact the structural remodeling observed in pressure overload hypertrophyor other diseases [44, 45].

Previous studies have shown transmural gradients in FA in normal hearts of sheep, human (in-vivo), and rat [24–26]. In sheep hearts, FA was lower in ENDO compared to MID or EPI regions. In the human study, FA was higher in MID than ENDO or EPI. Finally, in normal rat LV, a gradient of increasing FA from EPI to ENDO was observed [24]. We observed an opposite trend to that study, namely, with generally increasing values of FA from ENDO to EPI. Although Giannakidis et al. state that their result is consistent with measurements of myocyte CSA by McCrossan et al. [18], it is unclear how a *decrease* in L:W ratio would result in an *increase* in FA. Conversely, our data suggest a more logical correspondence between a *decrease* in FA when the L:W ratio *decreases*, as it does from EPI to ENDO (data not shown), however this correspondence did not reach statistical significance. In other words, diffusion would be more isotropic in voxels containing myocytes with a lower aspect ratio compared to more elongated myocytes. The same argument holds when comparing our results to those described by Campbell et al., who showed that in normal Sprague-Dawley rats, myocytes in the ENDO had larger CSA than EPI [16]. However, we did not observe a significant reduction of the gradient of FA in TAC, as one might expect from the results of a follow-up study [17]. Due to the similar transmural gradients in FA from DTI and MFA from confocal measurements, as well as similar definitions albeit from different sources, we expected there might be a correlation, however, this surprisingly did not reach statistical significance. This may be due to the somewhat limited study size, or perhaps the dip in FA near the epicardial surface, which might be a result of partial volume effects in that region.

To our knowledge, this is the first study to show correlations between DTI-derived parameters and myocyte geometry measurements in the same hearts. Others have used a similar approach to validate that the eigenvectors correspond with local helix and sheet orientations [21, 22], and more recently, that the eigenvalues correlate with collagen content [27, 46]. The correlations we observed between variables from DTI and measurements from histology were statistically significant, which may indicate that the DTI variables are influenced by myocyte geometry. Additionally, however, several of those same variables derived from DTI correlated significantly with the measured helix angle, which features a roughly linear transmural gradient. This may indicate that both myocyte geometry and some other structural features, which contribute to variations in DTI-derived parameters, also vary transmurally, giving rise to the moderate correlations we observed. In reality, the restriction to self-diffusion of water at the 100-μm scale is highly complex [47] so it is likely that structural features other than myocyte geometry (such as collagen content and composition) influence the diffusion weighted signal. However, we postulate that myocyte geometry plays an important role at the 100-μm resolution of our DTI scans. Potential methods to understand the contributions of different structural features to variation in DTI include simulation [48–50], and dPFG or mPFG pulse sequences or qMAS diffusion encoding [51].

Several limitations to this study are worth consideration. First, eight rats in each group is a relatively small number, and a better understanding of the differences between TAC and SHAM animals would require more. However, even with a relatively small N we were able to observe statistically significant, regionally varying differences in many DTI-derived parameters, as well as significant correlations with measurements from histological sections. Second, the region for correlating between DTI and histological measurement was limited to the LV free wall. This region was chosen because it is the most commonly studied. The nature of sectioning, staining, and imaging limits the expanse of regions that can be analyzed using this method. An interesting follow-up study would be to predict with a statistical degree of certainty myocyte geometry based on DTI in a different region, such as the septum. Third, due to the direction of sectioning for confocal imaging, we could not validate sheet angle measurements. However, this has previously been done and consistent agreement was found between DTI-derived sheet angles and those from histological measurement [20, 22] or synchrotron radiation imaging [52]. Lastly, all image data acquisition was performed on hearts fixed in the diastolic, unloaded state. The process of fixation alters the microstructural environment by cross-linking proteins, and tissue shrinkage is a known side-effect. Thus, 3D diffusivity may not be completely representative of that in vivo. Additionally, further shrinkage is known to occur during processing of samples for histology. Despite these structural changes in tissue structure, we observed significant correlations in multiple variables, indicating that the essence of tissue anisotropy and microstructure was preserved during fixation and histological processing.

## Conclusions

Regional variations in tissue microstructural features are evident in normal hearts, and remodeling during pressure overload hypertrophy is spatially non-uniform, with apparently more myocyte remodeling in MID and ENDO regions than EPI. Regional remodeling can be readily detected using cardiac DTI, with indications that parameters such as sheet angle dispersion and measures of anisotropy correlate significantly with variations in myocyte geometry, such as L:W ratio and MFA. As it is refined and developed further for in-vivo use in humans, cardiac DTI appears to be a promising method to non-invasively and comprehensively detect regional variations in cardiac tissue microstructure and its remodeling during disease.

## Data Availability

The datasets used and/or analysed during the current study are available from the corresponding author on reasonable request.

## Abbreviations

AoP: Aortic pressure
BW: Body weight
CL: Linear anisotropy
CP: Planar anisotropy
CS: Spherical anisotropy
CMR: Cardiovascular magnetic resonance
CSA: Cross-sectional area
DTI: Diffusion tensor imaging
ED: End-diastole
ENDO: Sub-endocardium
EF: Ejection fraction
EPI: Sub-epicardium
ES: End-systole
FA: Fractional anisotropy
HW: Heart weight
INS: Interventricular septum
L:H: Length:height ratio
L:W: Length:width ratio
LV: Left ventricle/left ventricular
MD: Mean diffusivity
MFA: Myocyte fractional anisotropy
MID: Midwall
SNR: Signal to noise ratio
TAC: Transverse aortic constriction
W:H: Width:height ratio
WGA: Wheat germ agglutinin

## References

[1] Benjamin EJ, Virani SS, Callaway CW, Chang AR, Cheng S, Chiuve SE (2018). Heart disease and stroke statistics—2018 update: a report from the American Heart Association. Circulation..

[2] Gerdes AM (1992). The use of isolated myocytes to evaluate myocardial remodeling. Trends Cardiovasc Med.

[3] Gjesdal O, Bluemke DA, Lima JA (2011). Cardiac remodeling at the population level--risk factors, screening, and outcomes. Nat Rev Cardiol.

[4] Bishop JE, Laurent GJ (1995). Collagen turnover and its regulation in the normal and hypertrophying heart. Eur Heart J.

[5] Bursac N (2014). Cardiac fibroblasts in pressure overload hypertrophy: the enemy within?. J Clin Invest.

[6] Conrad CH, Brooks WW, Hayes JA, Sen S, Robinson KG, Bing OHL (1995). Myocardial fibrosis and stiffness with hypertrophy and heart failure in the spontaneously hypertensive rat. Circulation.

[7] LeGrice IJ, Pope AJ, Sands GB, Whalley G, Doughty RN, Smaill BH. Progression of myocardial remodeling and mechanical dysfunction in the spontaneously hypertensive rat. Am J Physiol Heart Circ Physiol. 2012;303(11):1353–65. 10.1152/ajpheart.00748.2011 23001837

[8] Moore-Morris T, Guimarães-Camboa N, Banerjee I, Zambon AC, Kisseleva T, Velayoudon A (2014). Resident fibroblast lineages mediate pressure overload-induced cardiac fibrosis. J Clin Invest.

[9] Souders CA, Borg TK, Banerjee I, Baudino TA (2012). Pressure overload induces early morphological changes in the heart. Am J Pathol.

[10] Carruth ED, McCulloch AD, Omens JH (2016). Transmural gradients of myocardial structure and mechanics: implications for fiber stress and strain in pressure overload. Prog Biophys Mol Biol.

[11] Arts T, Reneman RS, Veenstra PC (1979). A model of the mechanics of the left ventricle. Ann Biomed Eng.

[12] Arts T, Veenstra PC, Reneman RS (1982). Epicardial deformation and left ventricular wall mechanics during ejection in the dog. Am J Phys.

[13] Bovendeerd PHM, Arts T, Huyghe JM, van Campen DH, Reneman RS (1992). Dependence of local left ventricular wall mechanics on myocardial fiber orientation: a model study. J Biomech.

[14] Guccione JM, Costa KD, McCulloch AD (1995). Finite element stress analysis of left ventricular mechanics in the beating dog heart. J Biomech.

[15] Leonard BL, Smaill BH, LeGrice IJ (2012). Structural remodeling and mechanical function in heart failure. Microsc Microanal.

[16] Campbell SE, Gerdes AM, Smith TD (1987). Comparison of regional differences in cardiac myocyte dimensions in rats, hamsters, and Guinea pigs. Anat Rec.

[17] Campbell SE, Korecky B, Rakusan K (1991). Remodeling of myocyte dimensions in hypertrophic and atrophic rat hearts. Circ Res.

[18] McCrossan ZA, Billeter R, White E (2004). Transmural changes in size, contractile and electrical properties of SHR left ventricular myocytes during compensated hypertrophy. Cardiovasc Res.

[19] Omens JH, Rodriguez EK, McCulloch AD (1996). Transmural changes in stress-free myocyte morphology during pressure overload hypertrophy in the rat. J Mol Cell Cardiol.

[20] Holmes AA, Scollan DF, Winslow RL (2000). Direct histological validation of diffusion tensor MRI in formaldehyde-fixed myocardium. Magn Reson Med.

[21] Hsu EW, Muzikant AL, Matulevicius SA, Penland RC, Henriquez CS (1998). Magnetic resonance myocardial fiber-orientation mapping with direct histological correlation. Am J Phys.

[22] Scollan DF, Holmes A, Winslow R, Forder J (1998). Histological validation of myocardial microstructure obtained from diffusion tensor magnetic resonance imaging. Am J Phys.

[23] Bernus O, Radjenovic A, Trew ML, LeGrice IJ, Sands GB, Magee DR (2015). Comparison of diffusion tensor imaging by cardiovascular magnetic resonance and gadolinium enhanced 3D image intensity approaches to investigation of structural anisotropy in explanted rat hearts. J Cardiovasc Magn Reson.

[24] Giannakidis A, Ferreira P, Gullberg GT, Firmin D, Pennell DJ (2015). Transmural gradients of preferential diffusion motility in the normal rat myocardium characterized by diffusion tensor imaging. J Cardiovas Magn Reson.

[25] Jiang Y, Guccione JM, Ratcliffe MB, Hsu EW (2007). Transmural heterogeneity of diffusion anisotropy in the sheep myocardium characterized by MR diffusion tensor imaging. Am J Physiol Heart Circ Physiol.

[26] McGill LA, Scott AD, Ferreira PF, Nielles-Vallespin S, Ismail T, Kilner PJ (2015). Heterogeneity of fractional anisotropy and mean diffusivity measurements by in vivo diffusion tensor imaging in normal human hearts. PLoS One.

[27] Abdullah OM, Drakos SG, Diakos NA, Wever-Pinzon O, Kfoury AG, Stehlik J (2014). Characterization of diffuse fibrosis in the failing human heart via diffusion tensor imaging and quantitative histological validation. NMR Biomed.

[28] Ferreira PF, Kilner PJ, McGill L-A, Nielles-Vallespin S, Scott AD, Ho SY (2014). In vivo cardiovascular magnetic resonance diffusion tensor imaging shows evidence of abnormal myocardial laminar orientations and mobility in hypertrophic cardiomyopathy. J Cardiovasc Magn Reson.

[29] Yamazaki KG, Romero-Perez D, Barraza-Hidalgo M, Cruz M, Rivas M, Cortez-Gomez B (2008). Short- and long-term effects of (−)-epicatechin on myocardial ischemia-reperfusion injury. Am J Physiol Heart Circ Physiol.

[30] McClymont D, Teh I, Carruth E, Omens J, McCulloch A, Whittington HJ (2017). Evaluation of non-Gaussian diffusion in cardiac MRI. Magn Reson Med.

[31] Teh I, McClymont D, Burton RAB, Maguire ML, Whittington HJ, Lygate CA (2016). Resolving fine cardiac structures in rats with high-resolution diffusion tensor imaging. Sci Rep.

[32] Teh I, Maguire ML, Schneider JE (2017). Efficient gradient calibration based on diffusion MRI. Magn Reson Med.

[33] Bensley JG, De Matteo R, Harding R, Black MJ (2016). Three-dimensional direct measurement of cardiomyocyte volume, nuclearity, and ploidy in thick histological sections. Sci Rep.

[34] Karlon WJ, Hsu P-P, Li S, Chien S, McCulloch AD, Omens JH (1999). Measurement of orientation and distribution of cellular alignment and cytoskeletal organization. Ann of Biomed Eng.

[35] Cox RW (1996). AFNI: software for analysis and visualization of functional magnetic resonance Neuroimages. Comp Biomed Res.

[36] Arsigny V, Fillard P, Pennec X, Ayache N (2006). Log-Euclidean metrics for fast and simple calculus on diffusion tensors. Magn Reson Med.

[37] Hales PW, JrE S, RAB B, Wright BJ, Bollensdorff C, Kohl P (2012). Histo-anatomical structure of the living isolated rat heart in two contraction states assessed by diffusion tensor MRI. Prog Biophys Mol Biol.

[38] Basser PJ, Pierpaoli C (1996). Microstructural and physiological features of tissues elucidated by quantitative-diffusion-tensor MRI. J Magn Res B.

[39] Ennis DB, Kindlman G, Rodriguez I, Helm PA, McVeigh ER (2005). Visualization of tensor fields using superquadric glyphs. Magn Reson Med.

[40] Kindo M, Gerelli S, Bouitbir J, Charles AL, Zoll J, Minh TH (2012). Pressure overload-induced mild cardiac hypertrophy reduces left ventricular transmural differences in mitochondrial respiratory chain activity and increases oxidative stress. Front Physiol.

[41] Mekkaoui C, Jackowski MP, Kostis WJ, Stoeck CT, Thiagalingam A, Reese TG (2018). Myocardial scar delineation using diffusion tensor magnetic resonance Tractography. J Am Heart Assoc.

[42] Nielles-Vallespin S, Mekkaoui C, Gatehouse P, Reese TG, Keegan J, Ferreira PF (2013). In vivo diffusion tensor MRI of the human heart: reproducibility of breath-hold and navigator-based approaches. Magn Reson Med.

[43] Welsh CL, DiBella EV, Hsu EW (2015). Higher-order motion-compensation for in vivo cardiac diffusion tensor imaging in rats. IEEE Trans Med Imaging.

[44] Croteau E, Benard F, Bentourkia M, Rousseau J, Paquette M, Lecomte R (2004). Quantitative myocardial perfusion and coronary reserve in rats with 13N-ammonia and small animal PET: impact of anesthesia and pharmacologic stress agents. J Nucl Med.

[45] Troalen T, Capron T, Bernard M, Kober F (2014). In vivo characterization of rodent cyclic myocardial perfusion variation at rest and during adenosine-induced stress using cine-ASL cardiovascular magnetic resonance. J Cardiovasc Magn Reson.

[46] Nielles-Vallespin S, Khalique Z, Ferreira PF, de Silva R, Scott AD, Kilner P (2017). Assessment of myocardial microstructural dynamics by in vivo diffusion tensor cardiac magnetic resonance. J Am Coll Cardiol.

[47] Wang L, Zhu Y-M, Li H, Liu W, Magnin IE (2011). Simulation of diffusion anisotropy in DTI for virtual cardiac fiber structure.

[48] Balls GT, Frank LR (2009). A simulation environment for diffusion weighted MR experiments in complex media. Magn Res Med.

[49] Baxter GT, Frank LR (2013). A computational model for diffusion weighted imaging of myelinated white matter. Neuroimage..

[50] Berry DB, Regner B, Galinsky V, Ward SR, Frank LR (2017). Relationships between tissue microstructure and the diffusion tensor in simulated skeletal muscle. Magn Reson Med.

[51] Szczepankiewicz F, Lasič S, van Westen D, Sundgren PC, Englund E, Westin CF (2015). Quantification of microscopic diffusion anisotropy disentangles effects of orientation dispersion from microstructure: applications in healthy volunteers and in brain tumors. Neuroimage..

[52] Teh I, McClymont D, Zdora MC, Whittington HJ, Davidoiu V, Lee J (2017). Validation of diffusion tensor MRI measurements of cardiac microstructure with structure tensor synchrotron radiation imaging. J Cardiovasc Magn Reson.

